# Assessment of a Districtwide Policy on Availability of Competitive Beverages in Boston Public Schools, Massachusetts, 2013

**DOI:** 10.5888/pcd13.150483

**Published:** 2016-03-03

**Authors:** Rebecca S. Mozaffarian, Steven L. Gortmaker, Erica L. Kenney, Jill E. Carter, M. Caitlin Westfall Howe, Jennifer F. Reiner, Angie L. Cradock

**Affiliations:** Author Affiliations: Steven L. Gortmaker, Erica L. Kenney, Jennifer F. Reiner, Angie L. Cradock, Harvard T.H. Chan School of Public Health, Boston, Massachusetts; Jill E. Carter, M. Caitlin Westfall Howe, Boston Public Schools Health & Wellness Department, Dorchester, Massachusetts.

## Abstract

**Introduction:**

Competitive beverages are drinks sold outside of the federally reimbursable school meals program and include beverages sold in vending machines, a la carte lines, school stores, and snack bars. Competitive beverages include sugar-sweetened beverages, which are associated with overweight and obesity. We described competitive beverage availability 9 years after the introduction in 2004 of district-wide nutrition standards for competitive beverages sold in Boston Public Schools.

**Methods:**

In 2013, we documented types of competitive beverages sold in 115 schools. We collected nutrient data to determine compliance with the standards. We evaluated the extent to which schools met the competitive-beverage standards and calculated the percentage of students who had access to beverages that met or did not meet the standards.

**Results:**

Of 115 schools, 89.6% met the competitive beverage nutrition standards; 88.5% of elementary schools and 61.5% of middle schools did not sell competitive beverages. Nutrition standards were met in 79.2% of high schools; 37.5% did not sell any competitive beverages, and 41.7% sold only beverages meeting the standards. Overall, 85.5% of students attended schools meeting the standards. Only 4.0% of students had access to sugar-sweetened beverages.

**Conclusion:**

A comprehensive, district-wide competitive beverage policy with implementation support can translate into a sustained healthful environment in public schools.

## Introduction

Competitive beverages are drinks sold outside of the federally reimbursable school meals program and include beverages sold in vending machines, a la carte lines, school stores, and snack bars. More than 90% of middle and high school students and 55% of elementary students in the United States have access to competitive beverages (1,2). Competitive beverage availability is associated with greater consumption of sugar-sweetened beverages (SSBs), such as soft drinks, fruit drinks, sweetened teas, and sports drinks (3–6), and consumption of SSBs is associated with overweight and obesity (7–9). Implementation and evaluation of policies to reduce availability or improve the quality of competitive beverages are needed.

Health and government organizations have identified competitive beverage standards and policies as important strategies for improving children’s health (10–12). As part of the Healthy, Hunger-Free Kids Act (HHFK), the US Department of Agriculture required that competitive beverages in all US schools that receive funds for the National School Lunch Program (95% of all US schools) meet nutrition standards in the 2014–2015 academic year (13). It is important to know whether such policies ensure that healthier competitive beverages are available.

Research on the effectiveness of district-level competitive beverage policy on availability has produced mixed findings. In 2 studies, SSBs were less likely to be available when prohibited by district policy (3,14), but in 2 other studies, no associations with decreased access to SSBs were found when policies did not prohibit all types (eg, banning soda only) (15,16). The sustained effects of competitive beverage policies and implementation at the district level are not well established.

In 2004, the Boston Public School (BPS) Committee approved a mandatory district-level policy for competitive beverage nutrition standards, including a ban on all SSBs. The standards have been in place for nearly a decade, but the extent to which the policy has been implemented and sustained is unknown. The objective of this study was to examine the level of compliance with the competitive beverage policy 9 years after its implementation. Our evaluation may help inform implementation of the HHFK Smart Snacks in Schools regulation as schools nationwide work toward achieving the nutrition standards. We hypothesized that most schools would meet the competitive beverage nutrition standards and that most BPS students would attend schools that meet the standards 9 years after implementation of the district-wide policy.

## Methods

We used a cross-sectional post-test study design to conduct an onsite audit of the availability of competitive beverages in all 122 BPS buildings from March through June 2013.

### Implementation of intervention on competitive beverage standards

The BPS district was the first in Massachusetts to develop district-level nutrition policy and standards for competitive foods and beverages (17). The competitive beverage standards were implemented in September 2004. During the 2004–2005 academic year, BPS food service directors worked with vendors to establish a 3-year bid cycle to change equipment and accommodate specified portion sizes. Circulars detailing the competitive beverage nutrition policy were posted on the BPS website for the staff and community. School principals were required to review circulars annually. BPS Food and Nutrition Services assumed responsibility of all vending contracts to ensure that vendors complied with the policy. In 2006, BPS developed a wellness policy on nutrition, physical activity, and other school-based activities that reinforces the competitive beverage standards. In 2007, a wellness coordinator was hired and tasked with ensuring competitive food and beverage policy implementation and technical assistance.

In 2010 and 2011, BPS health and wellness staff consulted with more than 400 individuals, including principals, teachers, school staff, parents and guardians, students, out-of-school coordinators, community partners, and national experts to inform the development of an implementation toolkit. The toolkit was a guide to encourage school staff, students, and families to create and support a cultural shift in the school food and beverage environment (17). It included sample family letters, flyers, posters, success stories, and strategies. The toolkit was distributed to schools through 2-hour professional development sessions offered monthly. Competitive food and beverage policy messages were incorporated into professional development sessions for physical education teachers, wellness council leaders, wellness champions, and health education teachers. An abbreviated toolkit was distributed to school principals at the beginning of each academic year. Customized training sessions were available on request and were delivered annually for new teachers, family and student engagement coordinators, and cafeteria managers. Health and wellness staff created a reporting system in which refresher trainings on the standards were provided when policy violations occurred. The standards were updated in 2010 to expand the scope to all foods and beverages sold, provided, or served to students on school property (including food trucks) or at school-sponsored events.

In 2013, when we conducted our evaluation, the competitive beverage nutrition standards did not allow the sale of SSBs, beverages containing artificial sweeteners, or beverages containing caffeine (except for chocolate milk, allowed because it contains only trace amounts of caffeine). In elementary schools, bottled water was the only competitive beverage allowed; 100% juices and milks were not permitted. Total sugars in flavored milk, allowed only in middle schools and high schools, were limited to 22 grams or fewer per 8-ounce serving, and only 8-ounce servings or less were allowed. Serving sizes of 100% fruit or vegetable juices were limited to 4 ounces in middle schools and 8 ounces in high schools (Box).

**Box. Boston Public School District Nutrition Standards for Competitive Beverages, 2013**

**Table:** 

Type of Beverage	Elementary Schools	Middle Schools	High Schools
Bottled water	Permitted at all grade levels. Noncarbonated, no additives, 0 mg sodium. (Drinking water from the tap or fountains is allowed and is not included in the competitive beverage standards.)		
100% Fruit or vegetable juice	Not permitted	≤4-oz portion size is permitted	≤8-oz portion size is permitted
Unflavored milk	Not permitted	≤8-oz portion size of 1% or nonfat milk only is permitted	≤8-oz portion size of 1% or nonfat milk only is permitted
Flavored milk	Not permitted	≤8oz portion size of nonfat milk only is permitted; must have ≤22 g total sugars	≤8-oz portion size of nonfat milk only is permitted; must have ≤22 g total sugars
Other beverages	Not permitted at any grade level		
Caffeine and artificial sweeteners	Not permitted at any grade level. Trace amounts of caffeine are allowed; chocolate milk has trace levels of caffeine and therefore is permitted.		

### Data collection

Trained research assistants used digital photographs and a standardized protocol to document beverages for sale at each access point in 122 BPS buildings. The standardized protocol was a paper-and-pencil survey developed by Harvard researchers. Data collectors documented the type and location of each access point, the type and size of each beverage, and the total number of slots for each beverage at each access point. Of the 122 BPS buildings surveyed, 7 school buildings were excluded because beverage standards did not apply to the populations served: 4 early child care and education centers, 1 adult technical academy, 1 counseling and intervention center, and 1 administrative building.

We scheduled visits to schools one week ahead of time. We defined an access point as any vending machine, a la carte cafeteria sale, school store, or cafe where competitive beverages were sold. We recorded the type, brand, flavor, portion size, percentage milk fat, and percentage juice for each unique beverage at each access point.

We collected nutrient and ingredient information for each beverage from manufacturer websites, including information on calories (kcal), total fat (g), sugar (g), and artificial sweetener or caffeine (yes/no). Beverages were classified into mutually exclusive categories: water, 100% juice (fruit or vegetable), unflavored milk, flavored milk, SSBs, diet drinks (zero calories with artificial sweeteners), or other (smoothies, coffee, unsweetened tea). We did not include the availability of competitive beverages in teacher’s lounges because researchers were unable to consistently access the lounges during school hours and because they were generally not accessible to students.

We used publicly available student enrollment data for each school for the 2012–2013 academic year to analyze demographics and to calculate the percentage of students with access to competitive beverages by grade level (18).

### Statistical analysis

We assessed the number and type of unique competitive beverages available at each access point and determined which met competitive beverage nutrition standards. We classified a school as having met the standards if every sold beverage met the standards or if none were sold. We classified schools that did not meet the nutrition standards into 2 categories: those that sold SSBs and those that did not sell SSBs. We made this distinction because the negative health effects of SSBs are more significant than the negative health effects of other beverages that do not meet standards (eg, 1% milk, 100% juice). For buildings that accommodated mixed grade levels (eg, elementary through high school), we applied the standards applicable to the lowest grade level. To determine the proportion of schools meeting the competitive beverage standards, we summed the number of schools that had no competitive beverages and the number of schools that fully met the standards and divided this sum by the total number of schools, stratified by grade level. We used logistic regression to determine whether beverages were more likely or less likely to meet the nutrition standards according to access point type.

We determined the percentage of students without access to any competitive beverages, the percentage of students with access only to competitive beverages meeting the standards, and the percentage of students with access to noncompliant beverages (further classifying noncompliant beverages as SSBs or non-SSBs). We then summed the number of students attending each school classified into each of these 4 categories and divided this number by the total number of students enrolled in BPS, stratified by grade level. We used SAS version 9.3 (SAS Institute Inc) to conduct all analyses. This study was determined to be exempt by the Harvard T.H. Chan School of Public Health Committee on Human Subjects.

## Results

Of the 115 school buildings included in analysis, 78 were elementary schools, 13 were middle schools, and 24 were high schools. Of the total population of 56,259 students, 40.8% were Hispanic and 36.7% were non-Hispanic black; 72.2% were eligible to receive free or reduced-price meals (Table 1).

**Table 1:** Characteristics of the 115 Schools Surveyed in the Boston Public School District, 2013^a^^,^ b

Characteristic	Elementary Schools (n = 78)	Middle Schools (n = 13)	High Schools (n = 24)	All Schools (n = 115)
**Total students enrolled, n**	31,848	6,866	17,545	56,259
**Average enrollment, n (SD)**	408.3 (194.8)	528.2 (280.8)	731.1 (555.4)	489.2 (336.3)
**Female, mean % (SD)**	47.5 (4.2)	45.2 (7.3)	47.9 (8.8)	47.3 (5.8)
**Free or reduced-price meals, mean % (SD)^c^**	70.6 (13.1)	80.9 (3.5)	72.5 (13.0)	72.2 (12.7)
**Race/ethnicity, mean % (SD)**				
Hispanic	43.3 (20.0)	36.9 (14.8)	34.9 (12.1)	40.8 (18.4)
Non-Hispanic black	32.6 (20.1)	45.8 (15.9)	45.2 (14.7)	36.7 (19.5)
Non-Hispanic white	15.4 (15.9)	6.6 (3.6)	10.8 (10.6)	13.5 (14.3)
Other	8.7 (9.2)	10.6 (14.1)	9.1 (8.0)	9.0 (9.5)

Abbreviation: SD, standard deviation.

a For school buildings accommodating mixed grade levels (eg, buildings with elementary through high school students), the standards were applicable to the lowest grade level.

b Seven school buildings were excluded from the analysis because the guidelines did not apply to the populations served. These 7 buildings included 4 early childcare and education centers, 1 technical academy, a counseling and intervention center, and the Food and Nutrition Services administrative building. Twenty-six elementary school buildings included kindergarten through 8th grade, and 1 included kindergarten through high school. Four middle school buildings included middle and high schools combined.

c Children from families with incomes ≤130% of the federal poverty level are eligible for free meals, and those with incomes between 130% and 185% of the poverty level are eligible for reduced‐price meals.

Of the 115 schools, 103 (89.6%) of schools met the competitive beverage nutrition standards; 86 (74.8%) sold no competitive beverages, and 17 (14.8%) sold only competitive beverage that met the standards. Elementary schools most frequently met the standards (93.6%), followed by middle schools (84.6%) and high schools (79.2%). Among elementary and middle schools, most met the standards because no competitive beverages were sold (88.5% of elementary schools and 61.5% of middle schools). Competitive beverages were sold at 47 access points in 29 schools. Most access points were vending machines (28 of 47 [59.6%]) or a la carte cafeteria sales (17 of 47 [36.2%]) (Table 2). We found no difference in whether or not competitive beverages met the standards by type of access point.

**Table 2:** Competitive Beverage Availability and Adherence to Competitive Beverage Nutrition Standards in 115 Boston Public Schools, by Grade Level, 2013

Category	Elementary Schools (n = 78)	Middle Schools (n = 13)	High Schools (n = 24)	All Schools (N = 115)
**Level of adherence, no. (%)**				
Schools met competitive beverage standards^a^	73 (93.6)	11 (84.6)	19 (79.2)	103 (89.6)
No competitive beverages sold	69 (88.5)	8 (61.5)	9 (37.5)	86 (74.8)
All competitive beverages sold met standards	4 (5.1)	3 (23.1)	10 (41.7)	17 (14.8)
Noncompliant competitive beverages sold, but not SSBs^b^	4 (5.1)	2 (15.4)	3 (12.5)	9 (7.8)
Noncompliant competitive beverages sold, including SSBs^b^^,^ c	1 (1.3)	0 (0.0)	2 (8.3)	3 (2.6)
**Total access points among schools selling competitive beverages, n**	10	8	29	47
Vending machines, n	4	5	19	28
A la carte cafeteria, n	6	2	9	17
Other^d^, n	0	1	1	2

Abbreviation: SSB, sugar-sweetened beverage.

a This category includes schools that met competitive beverage standards by not selling any competitive beverages or by selling only competitive beverages that met standards.

b SSBs are sugar sweetened beverages, including soft drinks, fruit drinks, sweetened teas, and sports drinks.

c May include other noncompliant beverages that were sold in addition to SSBs, such as low-fat milk or 100% juice.

d “Other” consists of 1 school store (middle school) and 1 café (high school).

Of the 12 schools in which competitive beverages were sold that did not meet the standards, 9 schools sold 100% juices, low-fat unflavored milk, or non-calorically sweetened water; 4 of these schools were elementary schools that sold 100% juice or low-fat milk (instead of nonfat milk). SSBs were sold in vending machines in 1 elementary school and 1 high school and as a la carte items in another high school (3 of 115 [2.6%] schools overall). Low-fat flavored milks were sold at 1 middle school and artificially sweetened water was sold at another middle school. Diet beverages were sold at 1 high school and tea, coffee, or fruit smoothies at another high school.

By percentage of students, most (88.4%) elementary school students and almost half of middle school students (44.9%) did not have any access to competitive beverages. Although 81.2% of high school students had access, most (56.6%) had access only to beverages that met the standards. Overall, 61.4% of students did not have access to any competitive beverages, and 24.1% had access only to competitive beverages that met standards, comprising 85.5% of the students district-wide (Figure). Only 4.0% of students had access to any SSBs, including 1.3% of elementary school students and 10.5% of high school students.

**Figure:**
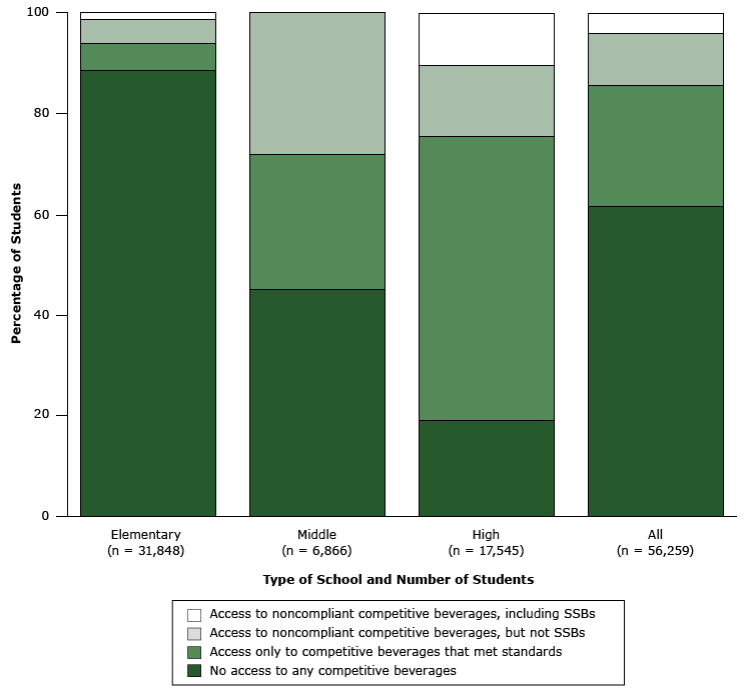
Access to competitive beverages among 56,259 students in 115 Boston Public Schools, 2013. **Table:** 

Type of School	No. of Students	No Access to Any Competitive Beverages, n (%)	Access Only to Competitive Beverages That Met Standards, n (%)	Access to Noncompliant Competitive Beverages, but not SSBs, n (%)	Access to Noncompliant Competitive Beverages, Including SSBs, n (%)
Elementary	31,848	28,159 (88.4)	1,801 (5.7)	1,489 (4.7)	399 (1.3)
Middle	6,866	3,083 (44.9)	1,835 (26.7)	1,948 (28.4)	0 (0)
High	17,545	3,307 (18.9)	9,925 (56.6)	2,476 (14.1)	1,837 (10.5)
All	56,259	34,549 (61.4)	13,561 (24.1)	5,913 (10.5)	2,236 (4.0)

## Discussion

Our study is a unique evaluation of a district-level competitive beverage policy 9 years after implementation. We provided evidence for sustainability and effectiveness in ensuring access to healthy competitive beverages. The overwhelming majority of Boston Public Schools (89.6%) were compliant with the competitive beverage nutrition standards. Most schools (74.8%) met the standards because no competitive beverages were sold, and 14.8% sold only competitive beverages that met the standards. District-wide, 85.5% of BPS students attended schools meeting the competitive beverage nutrition standards. Our study demonstrates that a comprehensive district-wide policy, in coordination with ongoing professional education, community-identified tools, and technical assistance training, can translate into a sustained healthier environment.

Nationwide, approximately 40% of district wellness policies ban soda as competitive beverages in schools, and fewer than 20% of elementary and 10% of middle schools or high schools ban other types of SSBs (19). Other nutrition standards may be included in wellness policies, but the provisions are often weak because they recommend that SSB availability should be limited or that they should be sold only to certain grade levels (20). In contrast, BPS competitive beverage policies were strong: SSB sales were banned at all venues and grade levels, and comprehensive supports and accountability measures were put into place to ensure compliance.

The BPS district succeeded in nearly eliminating the sale of SSBs, the beverages most strongly associated with negative health effects (7–9). Only 4% of students overall had access to SSBs, including 1.3% of elementary and 10.5% of high school students. These percentages are lower than national averages in 2013, when 12% of elementary school students, 63% of middle school students, and 88% of high school students had access to SSBs as competitive beverages (21). In our study, of the 12 schools in which competitive beverages were sold that did not meet the standards, 9 schools sold only 100% juices, low-fat unflavored milk, or non-calorically sweetened water, which are not associated with the same health risks as SSBs. The Massachusetts Competitive Beverage Guidelines (12) and HHFK Smart Snacks in Schools regulation (13) allow the sale of these beverages, and they may have been sold in the BPS district because of a lack of clarity about the standards.

Sustained adherence to the competitive beverage standards in the BPS district may have contributed to decreased consumption of SSBs among students. In 2006, two years after implementation of the ban on SSBs, the daily consumption of SSBs declined significantly among high school students (−0.30 servings per day), while no such decline was found nationally during that time (22). Nine years after implementation of the BPS beverage policy, data from the 2013 Youth Risk Behavior Surveillance System indicated that a smaller percentage of young people in Boston (16.8% [95% confidence interval, 14.0%–20.1%]; n = 1,157) than in 42 states (27.0% [95% confidence interval, 23.8%–30.5%]; n = 13,324) consumed one or more servings of SSB per day (23). Although these data are not longitudinal, they document SSB intake among students in Boston who spent all of their school years with the ban in effect. These values are consistent with evidence for the continuing impact of the policy. Other studies demonstrate that students consume fewer SSBs when school district policies prohibit them from being sold in schools (3,14,15). However, students may consume SSBs off campus after stringent standards are adopted, particularly if students have access to SSBs near their school (24).

This study has several strengths. First, we conducted an onsite audit with objective measures to document competitive beverages sold rather than relying on school staff reports, which are subject to recall and social desirability biases. We surveyed every school in the BPS district, providing a complete assessment of competitive beverage availability. We also provided detailed information on district-level competitive beverage policy implementation, a unique contribution to the scientific literature because details on implementation strategies are scarce. Finally, we conducted the assessment 9 years after the policy was implemented.

This study also has several limitations. One, we did not have baseline data on competitive beverage availability before policy was implemented. Thus, we do not know the extent to which competitive beverages were available before implementation, and we were unable to measure the magnitude of any changes in availability after implementation. However, the BPS school committee identified the need for a policy restricting the availability of competitive beverages, which suggests that competitive beverages were overly available and that standards were warranted. Two, we lacked a control group to exclude the possibility that adherence to the competitive beverage policy was part of a larger trend. However, national estimates indicate that SSBs are still widely offered as competitive beverages, and the availability of SSBs in the BPS district is well below national averages (21). Three, we did not determine the impact of the policy on revenue from competitive beverage sales, but increasing evidence demonstrates that strong competitive beverage standards are not associated with long-term revenue loss (25). Fourth, our findings may not be generalizable to all school districts nationwide; however, the BPS district serves predominantly racial/ethnic minority students from low-income households, and thus our findings may serve as an example for similar districts that could benefit from such policies and strategies, including implementation of Smart Snacks.

Nine years after implementation of a district-wide competitive beverage policy, 89.6% of schools met the standards, 85.5% of students attended schools in which the environment supported these standards, and only 4.0% of students had access to any SSBs. These findings and policy implementation strategies may be particularly encouraging to school districts nationally as they work to comply with HHFK Smart Snacks in Schools requirements (13). Our study demonstrates that a comprehensive district-wide competitive beverage policy with implementation support can translate into sustained healthier environments.
